# Association between Handgrip Strength and Periodontitis in Korean Adults Aged ≥30 Years: Data from the Korea National Health and Nutrition Examination Survey (2014–2015)

**DOI:** 10.3390/ijerph191710598

**Published:** 2022-08-25

**Authors:** Hye-Ryeong An, Jun-Seon Choi

**Affiliations:** 1 Department of Dental Hygiene, Graduate School, Gachon University, Incheon 21936, Korea; 2 Department of Dental Hygiene, College of Health Science, Gachon University, Incheon 21936, Korea

**Keywords:** community periodontal index, dental biofilm, hand function, handgrip strength, periodontitis, periodontal health, tooth brushing

## Abstract

This study used data from the Korea National Health and Nutrition Examination Survey 2014–2015 database to analyze the association between periodontitis and handgrip strength, a representative measure of hand impairment and function, in adults aged ≥30 years. The data of 5926 adults (male: 2766, females: 3160) who underwent handgrip strength and periodontal tissue examination and had neither rheumatoid arthritis nor osteoarthritis were analyzed. Handgrip strength was assessed using a digital grip strength dynamometer. The average values of the right handgrip strength, calculated separately by sex and age group (five 10-year age groups), were used as the cut-off for reduced handgrip strength. Periodontal status was evaluated using the Community Periodontal Index, defining scores ≥ 3 as periodontitis. Chi-square test and multivariate logistic regression analyses were performed to compare the differences in the prevalence of periodontitis according to handgrip strength. In the final regression model adjusted for risk factors for periodontitis, the likelihood of periodontitis decreased as the level of handgrip strength increased (*p* < 0.05). Therefore, this study suggests that handgrip strength may be a valuable indicator of periodontal health.

## 1. Introduction

According to the new classification scheme for periodontal and peri-implant diseases set by the European Federation of Periodontology and American Association of Periodontology in 2017, periodontitis has been subdivided into three categories based on pathophysiology: necrotizing periodontal diseases, periodontitis as a manifestation of systemic disease, and periodontitis, a category that combines the diseases previously recognized as “chronic” or “aggressive” [1]. Periodontitis is a chronic multifactorial inflammatory disease wherein periodontal tissues are destroyed by pathogenic bacteria such as *Porphyromonas gingivalis* and *Treponema denticola* colonizing in dental biofilm [2].

Periodontitis is mainly characterized by clinical attachment loss, radiographically assessed alveolar bone loss, presence of periodontal pockets, and gingival bleeding [1,3], and it is being monitored in many countries because it is the leading cause of tooth loss [4]. Furthermore, it is well-known that chronic inflammation in periodontal tissues and various systemic diseases, including cardiovascular disease, diabetes, pulmonary disease, and dementia, have a positive bidirectional relationship [5,6]. Hence, controlling inflammatory periodontal disease is essential for both oral and systemic health.

Dental biofilm accumulation on the surface of the teeth is the leading cause of periodontitis [2]. Biofilm is a sticky microbial community with more than 700 different bacterial species attached to salivary glycoproteins [2,7]. The most basic and effective method for the removal of dental biofilm is tooth brushing [2,8]. However, the effectiveness of manual tooth brushing generally depends on several factors, including brushing movements and the motor function of the hand [2,9,10]. In particular, impaired functions of the fingers or joints of the hand reportedly affect the extent of dental biofilm formation [11,12,13]. A cross-sectional study demonstrated that a decrease in handgrip strength and manual dexterity facilitated the accumulation of pathogenic dental biofilm in independent older adults [14].

Although the etiological factors of periodontitis have been elucidated, the disease does not show a significant decrease globally, unlike dental caries [15]. In particular, the prevalence of periodontitis requiring treatment is approximately 35% in Korean adults aged ≥30 years [16]. In addition, gingivitis and periodontitis were the most frequent causes of Korean adults visiting a medical institution between 2019 and 2020 [17]. Furthermore, hand function that can directly or indirectly affect the quality of oral care, especially in-hand manipulation skills, begin to decline in middle age [18]. Handgrip strength, indicating the force with which one can grip an object, peaks in the late 30s and decreases gradually thereafter [19,20,21,22]. Although the decline in hand function begins at a relatively early age, most previous studies that analyzed the correlation between oral health and hand function, including handgrip strength, were conducted only among the elderly. Therefore, there is a need to analyze the influence of hand function on periodontal status in both old and young adults at the age when the decline in hand function sets in. Hence, we analyzed the correlation between periodontitis and handgrip strength, a representative measure of hand impairment and function, in Korean adults aged ≥30 years using the Korea National Health and Nutrition Examination Survey (KNHANES) database.

## 2. Materials and Methods

### 2.1. Participants

This study used the KNHANES VI (2014–2015) database. The KNHANES, as a nationally representative survey conducted by the Korea Disease Control and Prevention Agency, is a surveillance system to evaluate the health and nutritional status of Koreans and monitor the prevalence and risk factors of major chronic diseases [16]. The KNHANES comprises health interviews, examinations, and nutrition surveys. Among the participants in KNHANES VI (2014–2015) (a total of 14,144 people), the study analyzed the data of subjects who met the following criteria: (1) age ≥30 years; (2) handgrip strength and periodontal tissue examination performed; (3) the right hand being the dominant hand; and (4) the absence of rheumatoid arthritis or osteoarthritis, which are diseases that affect handgrip strength. The data of 5926 participants (male: 2766, females: 3160) were analyzed. As a result of calculating the minimum sample size using G*power (ver. 3.1; Informer Technologies, Düsseldorf, Germany) [23], the study was confirmed to meet the required sample size (minimum sample size: 133 participants). Written informed consent was obtained from all participants prior to the KNHANES. KNHANES VI was approved by the Institutional Review Board (IRB) of the Korea Disease Control and Prevention Agency (IRB No. 2013–07CON-03–4C and 2013–12EXP-03–5C). The study protocol was approved by the IRB of Gachon University (IRB No.1044396–201910-HR-189–01), and all procedures were performed in accordance with the World Medical Association Declaration of Helsinki.

### 2.2. Measurements

The study used the data pertaining to sociodemographic characteristics (age, sex, personal income, and education), oral health behaviors (daily cigarette consumption, daily alcohol consumption, frequency of daily tooth brushing, use of interproximal cleaning devices, and dental check-ups in the past year), systemic health conditions (body mass index (BMI), glycated hemoglobin, total cholesterol (TC), triglyceride (TG), high-density lipoprotein cholesterol (HDL-C), and low-density lipoprotein cholesterol (LDL-C)), hand function (handgrip strength), and periodontal status (Community Periodontal Index (CPI)). Oral health behaviors were reclassified as follows based on previous studies [16,24,25]: daily cigarette consumption (non-smoker, light smoker (<10 cigarettes), and moderate/heavy smoker (≥10 cigarettes)), daily alcohol consumption (non-drinker, light drinker (0–6 units for men or 0–2 units for women), moderate/heavy drinker (≥7 units for men or ≥3 units for women); the study only considered the amount of alcohol regardless of the type), frequency of daily tooth brushing (≤2 and ≥3 times), dental check-ups in the past year (yes/no), and use of interproximal cleaning devices (yes/no). BMI was classified as underweight (<18.5 kg/m^2^), normal (18.5–24.9 kg/m^2^), and obese (≥25 kg/m^2^), and glycated hemoglobin was classified as normal (<6.5%) and diabetes (≥6.5%) [26]. TC, TG, HDL-C, and LDL-C were considered normal at levels <200, <150, ≥60, and <130 mg/dL, respectively [27]. Handgrip strength was measured to assess hand function. Low handgrip strength indicates a decline in the overall function [28]. Handgrip strength was measured using a digital grip strength dynamometer (T.K.K 5401; Takei, Tokyo, Japan) that can measure a force of 5.0 kg to 100.0 kg in 0.1 kg increments. The participants were asked to stand upright with their feet hip-width apart and elbows fully extended. The handle of the dynamometer was adjusted such that the second joint of the index finger was flexed at 90°. The participants were instructed to squeeze the grip with the maximum force for at least three seconds and not to shake the hand dynamometer during the measurements.

The normal range of handgrip strength differs greatly according to sex and age [21]. Therefore, to set the cut-off values for defining reduced handgrip strength, the average value of the right handgrip strength was calculated separately by sex and age group (five groups, ranging from 30 years to above 70 years of age). All participants were categorized into two groups based on the average handgrip strength of their respective groups. Participants whose handgrip strength was greater than or equal to the average were classified into the normal group, and those whose handgrip strength was lower than the average were classified into the reduced group. The average values (M ± SD) of handgrip strength by sex and age group were as follows: males (30s: 44.79 ± 0.36 kg, 40s: 43.20 ± 0.30 kg, 50s: 40.08 ± 0.27 kg, 60s: 37.15 ± 0.30 kg, and over 70 years: 30.88 ± 0.35 kg) and females (30s: 26.00 ± 0.20 kg, 40s: 25.89 ± 0.19 kg, 50s: 24.83 ± 0.20 kg, 60s: 22.96 ± 0.23 kg, and over 70 years: 19.03 ± 0.30 kg). Periodontal status was evaluated using the CPI [29]. The mouth was divided into six parts: three parts in the maxillary arch (right posterior (#18–14), anterior (#13–23), and left posterior (#24–28)) and three parts in the mandibular arch (right posterior (#48–44), anterior (#43–33), and left posterior (#34–38)). A dentist who completed the calibration training examined the index teeth (#16, #17, #11, #26, #27, #31, #36, #37, #46, or #47) with a CPI probe. Each index tooth was evaluated for gingival bleeding, calculus, and periodontal pockets and scored on a scale of 0–4: healthy periodontal tissue (CPI 0), gingival bleeding on probing (CPI 1), periodontal tissue with gingival calculus (CPI 2), periodontal tissue with shallow periodontal pockets (4–5 mm) (CPI 3), and periodontal tissue with deep periodontal pockets (≥6 mm) (CPI 4) [16]. Those with a CPI score less than 3 were classified into the non-periodontitis group, whereas those with a CPI score of 3 or higher were classified into the periodontitis group [16].

### 2.3. Statistical Analysis

The collected data were analyzed using SPSS Statistics software (ver. 23; IBM Co., Armonk, NY, USA). A chi-square test was used to compare the differences in the prevalence of periodontitis according to sociodemographic characteristics, oral health behaviors, systemic health conditions, and handgrip strength. After adjusting for potential confounders, multivariate logistic regression analyses were performed to determine the strength of association between handgrip strength and periodontitis. For the multivariate logistic regression analyses, variables with *p*-value < 0.05 in the chi-square test were included as independent variables, and periodontitis was the dependent variable. Statistical significance was set at α = 0.05.

## 3. Results

### 3.1. Periodontitis According to Sociodemographic Characteristics and Oral Health Behaviors

The prevalence of periodontitis was higher in men (40.8%) than in women (27.9%) and increased with age, with the highest rate in the age group of 60–69 years (50.0%) (*p* < 0.001). The prevalence of periodontitis was higher in the group with the lowest personal income (39.8%) and in those with an educational level of primary school completion (50.9%) than those in the respective groups when compared (*p* < 0.001). The prevalence of periodontitis was higher in heavy smokers (47.6%), those who brushed their teeth twice or less per day (39.2%), those who had not undergone dental check-ups in the previous year (36.1%), and those who did not use interproximal cleaning devices (40.5%) than that in subjects in the respective groups when compared (*p* < 0.01) (Table 1).

### 3.2. Periodontitis According to Systemic Health Conditions and Handgrip Strength

The prevalence of periodontitis was higher in those with a BMI of ≥25 kg/m^2^ (42.0%), glycated hemoglobin level of ≥6.5% (55.5%), TC level of ≥240 mg/dL (39.8%), TG level of ≥200 mg/dL (45.0%), and HDL-C level of <40 mg/dL (44.5%) than it was in subjects in the respective groups when compared (*p* < 0.05). In addition, the prevalence of periodontitis was higher in the reduced handgrip strength group (38.3%) than in the normal handgrip strength group (33.1%) (*p* = 0.001) (Table 2).

### 3.3. Strength of Association between Periodontitis and Handgrip Strength

In the crude model in which handgrip strength was input as a continuous variable, handgrip strength and periodontitis showed a negative correlation (confidence interval (CI): 0.984–0.997). Moreover, in the multivariate logistic regression model adjusted for the risk factors of periodontitis, the likelihood of periodontitis decreased as the handgrip strength increased (CI: 0.974–0.999) (Table 3).

## 4. Discussion

Although bacterial biofilm on dental surfaces is the primary cause of periodontitis, several factors, including systemic health, are involved in its progression [30]. Therefore, for further prevention of periodontitis, various systemic factors other than intraoral factors need to be considered. Hand function has been known to be associated with personal hygiene, especially oral health behaviors in older adults [11,13,31,32]. However, hand function, including in-hand manipulation skills and handgrip strength, peaks in the 30s and then declines continuously with age [18,20,21,22]. Accordingly, it is necessary to determine the effect of hand function on periodontal health and include middle-aged adults in the studies. Hence, this study analyzed the association between periodontitis and handgrip strength in 5926 Korean adults aged ≥30 years who participated in KNHANES VI (2014–2015). Handgrip strength represents not only hand impairment and function but also overall muscle strength [33,34]. More importantly, it is a useful tool that can indirectly evaluate the systemic health status of the adult population, as poor handgrip strength is closely related to diseases that contribute to mortality, such as cardiovascular disease and cancer [35,36].

Bivariate analysis revealed that the proportion of patients with reduced handgrip strength was higher in the periodontitis group than in the normal group (*p* = 0.001). Moreover, in the final regression model adjusted for the risk factors of periodontitis and considering handgrip strength as a continuous variable, it was confirmed that the higher the handgrip strength, the lower the incidence of periodontitis (*p* = 0.030). Thus, this study suggests that reduced handgrip strength can be a predictor of periodontitis in adults aged ≥30 years. Several previous studies, which included mostly elderly subjects, have reported the importance of hand function in the quality of oral care [11,32]. One study reported that older adults with decreased manual dexterity or handgrip strength had a higher accumulation of mature dental biofilm, a causative factor of oral diseases [32]. Hashimoto et al. [37] suggested that older adults aged 80 years or above who showed high handgrip strength had more teeth than did those with low handgrip strength. In addition, cross-sectional studies among adults aged ≥19 years reported that people with low handgrip strength showed poorer oral health behaviors, including tooth brushing [38,39]. Therefore, considering previous studies and our findings, we infer that reduced handgrip strength has a direct and indirect adverse effect on the ability to perform daily oral care in adults aged ≥30 years and eventually contributes to developing periodontal inflammation over a prolonged period. Low handgrip strength can cause discomfort and premature fatigue of the hand when manipulating a toothbrush handle. Fatigue, in turn, reduces the brushing time, brushing force, and brushing movements, eventually resulting in less efficient biofilm removal. Therefore, interventional programs for handgrip strength enhancement can improve hand motor skills and periodontal health. Customized oral hygiene care programs should also be provided according to individual handgrip strength. In particular, to perform a rolling stroke that is generally recommended for adults in South Korea, the performer must turn the wrist after holding the toothbrush handle with the palm of the hand [40]. This means that when using the rolling stroke method, biofilm removal may be more affected by hand function than it would when using other brushing techniques. Hence, dental hygienists need to continuously motivate and support adults with low handgrip strength to provide optimal oral hygiene care. In addition, considering previous studies finding that probiotics or paraprobiotics included in chewing gum or toothpaste significantly reduce specific periodontopathogens related to periodontitis and maintain a balanced oral microbiota, ultimately leading to improvement quality of clinical activities performed by professionals and self-oral care performed by patients [41,42], these agents can be recommended to periodontal patients with reduced handgrip strength as a supplement to improve periodontal health. Finally, a collaboration between dental professionals and musculoskeletal specialists is necessary for optimal periodontal health.

This study is of significance, as it is the first publication to demonstrate the association between reduced handgrip strength and periodontitis in adults aged ≥30 years, using the KNHANES database, which represents the health status of the South Korean population. In addition, although reduced handgrip strength was not found to be a predisposing factor for periodontitis, the study has provided an opportunity to understand that handgrip strength should be included among the management items for periodontal health.

However, this study has several limitations. First, as the KNHANES is a cross-sectional survey, it is difficult to explain the causal relationships between the variables used in the study, especially between periodontitis and reduced handgrip strength. Second, we considered the average handgrip strength, which was calculated separately by sex and age group, as the cut-off value for defining reduced handgrip strength, and handgrip strength was used as a continuous variable in the logistic regression model. However, according to several previous studies, the abnormal range of handgrip strength varies depending on various factors, including ethnicity and sex [22]. Consequently, the degree of association between periodontitis and reduced handgrip strength may differ depending on the cut-off value used to define the reduced handgrip strength. Third, the CPI score used to evaluate periodontal status may not accurately reflect the periodontal status of all existing teeth because only the index teeth were examined to represent each sextant [43]. Fourth, although the final regression model was adjusted for the risk factors of periodontitis, variables that we did not consider might have affected our results. In particular, tooth-brushing techniques and duration are important factors for biofilm removal [2,44]; however, these habit-related factors were not considered in this study. Hence, future studies should use a longitudinal design and adjust for multiple confounding variables that affect periodontal status to determine the association between handgrip strength and periodontitis.

## 5. Conclusions

Analyzing the association between handgrip strength and periodontitis using the KNHANES database that represents the health status of the South Korean population confirmed that reduced handgrip strength was independently associated with periodontitis in adults aged ≥30 years. Therefore, the study’s findings suggest that maintaining stronger handgrip strength may contribute to improving periodontal health. Additionally, customized oral hygiene care programs should be provided depending on the individual’s handgrip strength.

## Figures and Tables

**Table 1:** Association between sociodemographic characteristics or oral health behaviors and periodontitis.

Characteristics	Division	*N*	Periodontal Status		χ^2^ (*p*)
			Non- Periodontitis	Periodontitis	
Socio-demographic characteristics					
Sex	Male	2766	1550 (59.2)	1216 (40.8)	107.770 (<0.001)
	Female	3160	2228 (72.1)	932 (27.9)	
Age (years)	30–39	1271	1070 (84.4)	201 (15.6)	488.095 (<0.001)
	40–49	1345	956 (69.2)	389 (30.8)	
	50–59	1404	752 (52.1)	652 (47.9)	
	60–69	1095	560 (50.0)	535 (50.0)	
	≥70	811	440 (53.2)	371 (46.8)	
Personal income	Lowest (<25%)	1325	787 (60.2)	538 (39.8)	37.989 (<0.001)
	Middle low (25–50%)	1464	891 (63.0)	573 (37.0)	
	Middle high (50–75%)	1554	1039 (68.1)	515 (31.9)	
	Highest (>75%)	1560	1049 (69.8)	511 (30.2)	
Education	Primary school	1114	563 (49.1)	551 (50.9)	258.044 (<0.001)
	Middle school	689	363 (51.6)	326 (48.4)	
	High school	1922	1215 (63.8)	707 (36.2)	
	≥College	2165	1615 (75.5)	550 (24.5)	
Oral health behaviors					
Daily cigarette	Non-smoker	4728	3158 (69.0)	1570 (31.0)	111.428 (<0.001)
Consumption ^†^	Light smoker	229	133 (60.4)	96 (39.6)	
	Heavy smoker	905	450 (52.4)	455 (47.6)	
Daily alcohol	Non-drinker	1608	995 (63.7)	613 (36.3)	3.672
Consumption ^††^	Light drinker	2609	1687 (66.6)	922 (33.4)	(0.247)
	Moderate/heavy drinker	1648	1061 (65.1)	587 (34.9)	
Frequency of daily tooth brushing	≤2	2737	1619 (60.8)	1118 (39.2)	50.251
	≥3	3060	2091 (69.7)	969 (30.3)	(<0.001)
Dental check-ups in the past year	Yes	1897	1278 (68.7)	619 (31.3)	13.200
	No	3962	2463 (63.9)	1499 (36.1)	(0.002)
Use of interproximal cleaning devices	Yes	2125	1567 (75.1)	558 (24.9)	149.812
	No	3736	2175 (59.5)	1561 (40.5)	(<0.001)

%, weighted. ^†^ Light smokers (<10 cigarettes); moderate/heavy smokers (≥10 cigarettes). ^††^ Light drinkers (<7 units for men or <3 units for women); moderate/heavy drinkers (≥7 units for men or ≥3 units for women).

**Table 2:** Association between systemic health conditions or handgrip strength and periodontitis.

Characteristics	Division	*N*	Periodontal Status		χ^2^ (*p*)
			Non- Periodontitis	Periodontitis	
BMI	Underweight (<18.5 kg/m^2^)	194	145 (78.9)	49 (21.1)	75.008 (<0.001)
	Normal (18.5–24.9 kg/m^2^)	3782	2513 (68.2)	1269 (31.8)	
	Obese (≥25 kg/m^2^)	1913	1086 (58.0)	827 (42.0)	
Glycated	Normal (<6.5%)	5018	3305 (67.5)	1713 (32.5)	103.632 (<0.001)
hemoglobin	Diabetes (≥6.5%)	559	261 (44.5)	298 (55.5)	
TC	Optimal/normal (<200 mg/dL)	3466	2239 (66.9)	1227 (33.1)	10.408 (0.015)
	Borderline high (200–239 mg/dL)	1635	1041 (64.3)	594 (35.7)	
	Abnormal/high (≥240 mg/dL)	499	297 (60.2)	202 (39.8)	
TG	Optimal/normal (<150 mg/dL)	3849	2596 (69.7)	1253 (30.3)	93.061 (<0.001)
	Borderline high (150–199 mg/dL)	780	453 (59.3)	327 (40.7)	
	Abnormal/high (≥200 mg/dL)	971	528 (55.0)	443 (45.0)	
HDL-C	Reasonably good (≥60 mg/dL)	1210	888 (75.5)	322 (24.5)	108.906 (<0.001)
	Normal (40–59 mg/dL)	3166	2025 (65.5)	1141 (34.5)	
	Abnormal/low (<40 mg/dL)	1224	664 (55.5)	560 (44.5)	
LDL-C	Normal (<130 mg/dL)	2268	1419 (65.1)	849 (34.9)	3.291 (0.292)
	Borderline high (130–159 mg/dL)	738	462 (63.7)	276 (36.3)	
	Abnormal high (≥160 mg/dL)	300	179 (59.9)	121 (40.1)	
Handgrip	Normal	3998	2616 (66.9)	1382 (33.1)	15.083
strength	Reduced/poor	1928	1162 (61.7)	766 (38.3)	(0.001)

%, weighted. BMI, body mass index; TC, total cholesterol; TG, triglyceride; HDL-C, high-density lipoprotein cholesterol; LDL-C, low-density lipoprotein cholesterol.

**Table 3:** Factors associated with periodontitis based on multiple logistic regression analysis.

Characteristics	Crude		Adjusted ^†^	
	OR (95% CI)	*p*	OR (95% CI)	*p*
Handgrip strength (continuous)	0.990 (0.984–0.997)	0.002	0.986 (0.974–0.999)	0.030

CI, confidence interval. *p*-values were obtained from multivariate logistic regression analyses. ^†^ Adjusted for sociodemographic characteristics (age, sex, personal income, education), oral health behaviors (daily cigarette consumption, frequency of tooth brushing, dental check-ups in the past year, use of interproximal cleaning devices), and systemic health conditions (glycated hemoglobin, BMI, TC, TG, HDL-C).

## Data Availability

The findings of this study were analyzed using a publicly available database. The data are located at: https://knhanes.kdca.go.kr/knhanes/sub03/sub03_02_05.do. (accessed on 2 May 2021).
